# Assessment of water quality of the Halda River using multiple WQI approaches: Implications for Riverine Ecosystem and Sustainable Management

**DOI:** 10.1371/journal.pone.0350672

**Published:** 2026-06-18

**Authors:** Ahasanul Karim, Muhammad Towhid Moula, Ranjit K. Nath, Masud Rana Rashel, Mh. Mosfeka Chowdhury, Abu Mansur, Md. Masudur Rhaman, Mayeen Uddin Khandaker

**Affiliations:** 1 Department of Chemistry, Faculty of Science and Technology, Chittagong University of Engineering and Technology (CUET), Chattogram, Bangladesh; 2 Department of Chemistry, Fatikchari Government College, Chattogram, Bangladesh; 3 Instrumentation and Control Lab, Department of Mechatronics Engineering, University of Evora, Évora, Portugal; 4 Department of Chemistry, Nazirhat College, Chattogram, Bangladesh; 5 Chattogram Water Supply and Sewerage Authority, Chattogram City Corporation, Chattogram, Bangladesh; 6 Department of Chemistry, Wayne State University, Detroit, Michigan, United States of America; 7 Applied Physics and Radiation Technologies Group, CCDCU, Faculty of Engineering and Technology, Sunway University, Bandar Sunway, Selangor, Malaysia; 8 Department of Physics, College of Science, Korea University, Seoul, Republic of Korea; 9 Faculty of Graduate Studies, Daffodil International University, Dhaka, Bangladesh; Amity University Amity Institute of Biotechnology, INDIA

## Abstract

The Halda River-Bangladesh’s only natural breeding ground for major carps and a vital freshwater resource is undergoing alarming deterioration in water quality. This study assessed its ecological condition by applying seven Water Quality Indices (WQIs): Weighted Arithmetic, British Columbia, Canadian, Malaysian, Oregon, Assigned, and the recently developed Method of the Removal Effects of Criteria (MEREC-WQI). To record the seasonal and regional changes, surface water samples were collected during both dry and wet seasons. It has been observed that the respective values of several important parameters like turbidity, ammonia, biochemical oxygen demand (BOD₅), and dissolved oxygen (DO), often exceeded national reference limits or guidelines. The deduced water quality index (WQI) scores ranged from 25 to 45, which categorized the river water as ‘poor’ to ‘very poor’. The water quality shows more deterioration during the dry season, which might be related to the reduction of water flow, industrial discharges, etc. Overall, the uses of various WQI techniques help to obtain a comprehensive and comparative assessment of river health. This study indicates a non-negligible man made stress on the Halda River ecosystem, and it recommends to implement a comprehensive pollution control strategies and viable watershed management to protect the River’s ecological balance.

## 1 Introduction

Surface waters of various sources including river, canal, ponds, lakes, etc. facilitate numerous activities including drinking, agriculture, sanitation, industrial activities for sustenance of lives; however, those water sources receive significant contamination from industrial effluents, agricultural runoff, rapid urbanization, etc. [1,2]. This situation is especially significant in developing countries like Bangladesh. According to the World Health Organization (2016), almost 159 million peoples worldwide are using the contaminated surface water to meet their daily necessities [3–5].

The Halda River, located in South-Eastern of Bangladesh is serving as the sole natural breeding habitat for major carps in South Asia, hence, possesses significant ecological and social importance among the numerous rivers in Bangladesh. Particularly, this river facilitates many fishing households and shows rich biodiversity; however, due to the rapid and unregulated urbanization and industrial settings in the vicinity of the river, agricultural runoff, substantial effluent discharges from untreated industrial and municipal solid waste, etc. pose a non-negligible threat to the delicate aquatic ecosystems of this river [6–9] as well as posing potential economic repercussions for the national fisheries industry [6,7,10].

Considering the significant contribution of this river to national economy, numerous studies were performed on different aspects of this river’s hydrology, ecology, human-driven degradation. However, most of those studies focused only on few hydrological features, without emphasizing the ecological conditions that are important for successful carp spawning [11]. Note that the overall water quality and associated factors like dissolved oxygen (DO), pH, temperature, free CO₂, and alkalinity directly affect spawning success and larval development (i.e., the fish growth and reproduction) [12–15]. Recognizing this situation, several studies on some other major Bangladeshi rivers, including the Karnaphuli, Shitalakshya, Dhaleshwari, were performed for various water quality indices (WQIs) such as Canadian WQI (CWQI), Weighted Arithmetic WQI (WAWQI), and Assigned WQI (AWQI); all of which indicates the declining water quality of those rivers due to the major reasons such as industrial effluents, municipal wastewater, agricultural runoff [10,16–18]. On the other hand, majority of those studies are confined to one or two indices but not considered the index comparability or interpretability across various river systems. It is worth mentioning that the WQIs vary in their design and objectives worldwide, hence shows contradictory classifications even utilized on same datasets [19–21]. Note that some WQIs such as Malaysian WQI (MWQI) and AWQI were developed for tropical environments, whereas British Columbia WQI (BCWQI) and Oregon WQI (OWQI) were formulated for temperate systems; this fact indicates the justification of their applicability to rivers in tropical countries like the Halda, which fulfill distinct biological roles. Furthermore, each WQI possesses intrinsic limitations such as subjectivity in parameter weighting, regional dependence, restricted ecological context, etc., which highlights the necessity for multi-index or comparison methodologies to obtain more reliable and significant evaluations [22–25].

Although over thirty articles have examined the water quality of Halda River, most of them are limited by narrowing the temporal and spatial coverage, selectively focused on a few locations and short observation periods, and utilized only a singular WQI [26,27]. Therefore, it can be mentioned that no thorough investigation has yet to be evaluated various global WQIs of the Halda River, which relates the findings to ecological thresholds for carp spawning, or analyzed the results considering the framework of UN Sustainable Development Goals (SDGs) [28].

This study aims to provide the first integrated water quality assessment of the Halda River using a range of WQIs, including WAWQI, BCWQI, CWQI, AWQI, MWQI, OWQI, and RPI-across two major seasons (dry and wet) for examining the variations in pollution levels and health of the river. The characteristics of land use and the pollution scenario along the river continuum were considered to categorize the sampling sites into three types: upstream rural areas with little disturbance, midstream sections impacted by urban and small-scale industrial activities, and downstream areas vulnerable to rising domestic and industrial waste [29]. Since the mid- and downstream regions are ideal for carp breeding grounds, where the decline in water quality poses the biggest risk to reproduction, this design exhibits ecological sensitivity [26,30]. Some parameters such as dissolved oxygen (DO), turbidity, ammonia, and biochemical oxygen demand (BOD₅) have direct effects on fish spawning, egg development and larval survival, therefore, these were chosen for study following the ecological and regulatory priorities (EQS, 1997; DoE guidelines) [26–28].

In this work, the introduced multi-index evaluation of water quality facilitates the global WQI framework for tropical and environmentally sensitive areas like the Halda River [29,30–32]. The obtained results may be helpful for the sustainable management of the ecologically and nationally significant Halda river as well as the results may enhance the global conversation on water quality evaluation for developing nations. In addition, this work aligns with several sustainable development goals like SDGs 6, 14 and 15 [31,32].

With the aid of specific water quality indices like WAWQI or CWQI, the water quality of the major rivers of Bangladesh has been evaluated by several studies. Yet, comparative studies employing specific water quality indices according to the guidelines of international water quality indices are scarce, particularly for water bodies like the Halda River, which are sensitive from the perspective of natural environments. There is a scarcity of comprehensive water quality assessment through the application of multiple water quality indices, seasonal variations, and regional gradients, as most of the earlier studies conducted on the Halda River were focused on specific physicochemical aspects [33,34].

## 2 Methodology

### 2.1 Study area

The Halda River is a river in southeastern Bangladesh. The river is part of the Chittagong Hill Tracts. The river flows through Khagrachari and Chattogram districts. The river then flows into the Karnaphuli River. The watershed area includes a mix of various land use practices such as agricultural areas, rural settlements, and urbanization [33,35].

The Halda River, extending roughly 81 kilometers, has originated mainly from the Batnatali Union of Manikchari Upazila, where the Salda spring acts as its source [33]. It then passes through the Khagrachari and Chittagong districts and finally meet the Bay of Bengal. During the rainy season (May–August), this river attains highest levels of water, sometime overflooded the river bank, while on the other seasons the water levels are relatively low and low current. The water levels generally oscillate within 5–6 meters following the seasonal variations, and this fact indicates a considerable hydrological variability between the dry and rainy seasons [36,37].

**Fig 1:**
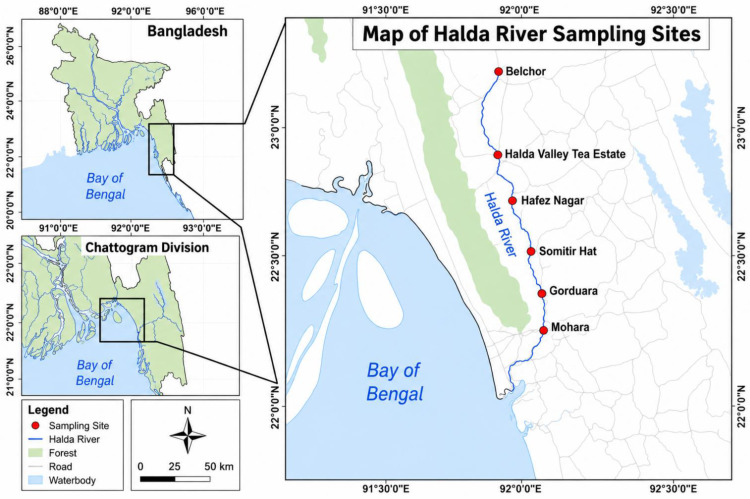
Map of Halda River sampling sites in Bangladesh. The map was created by the authors using QGIS (v3.40) based on Natural Earth datasets (https://www.naturalearthdata.com; public domain/CC BY 4.0). No copyrighted or third-party proprietary materials were used.

A total of six locations or segments were chosen in between Patachora and Kalurghat along the river stretch to ensure the representative coverage of diverse land use, ecological sensitivity, hydrological and anthropogenic effects (Fig 1). These segments can be identified mainly as upstream rural regions, midstream semi-urban areas, and downstream industrial sectors [33,38].

The authors generated the research area map of the Halda River utilizing QGIS (Version 3.40) and publicly accessible Natural Earth datasets (administrative boundaries, rivers, and lakes; CC BY 4.0). No proprietary or copyrighted images were utilized.

### 2.2 Sample collection permission statements

Field sampling was conducted in the Halda River, a natural, publicly accessible waterway. No specific permission was required for sample collection because the study sites were not privately owned or part of a designated protected area. The sampling did not involve endangered or protected species, and the work strictly adhered to institutional guidelines and relevant national regulations for environmental research.

### 2.3 Data collection

Physicochemical parameters were measured following the *Standard Methods for the Examination of Water and Wastewater* [39] and the Department of Environment (DoE) protocols [28]. The analyzed parameters included temperature, pH, electrical conductivity (EC), total dissolved solids (TDS), total suspended solids (TSS), turbidity, dissolved oxygen (DO), biochemical oxygen demand (BOD₅), chemical oxygen demand (COD), calcium, ammonia, and chloride.

In the year 2023, the water samples were collected in both the wet (April–September) and dry (December–February) seasons. Pre-acid-washed polyethylene bottles were used to contain the collected water from a depth of 0.5 m with a Van Dorn sampler to minimize surface contamination. To ensure the accuracy of nutrient and BOD₅/COD analyses, several samples were preserved in iceboxes and then transported to the laboratory within few hours of collection. For the purpose of cation analysis, some other collected samples were acidified to pH < 2 using concentrated nitric acid (HNO₃) to prevent precipitation and adsorption losses.

In total, 144 samples were analyzed (12 parameters × 6 sites × 2 seasons). Based on 20 paired observations, the regression analysis yielded strong correlations (R² = 0.92 for the wet season and R² = 0.87 for the dry season) between turbidity and TSS. Consequently, the following empirical equations adapted from were applied to estimate TSS from turbidity measurements [40]:


$$\begin{array}{c} {\text{TSS}}_{\text{wet}}\;=\text{ 0}.\text{86 turbidity }+\text{ 9}.\text{99} \end{array}$$ (1)

$$\begin{array}{c} {\text{TSS}}_{\text{dry}}=0.79\text{ turbidity }+\;4.36 \end{array}$$ (2)

Where TSS_wet_ is the wet season total suspended solids (mg/L), TSS_dry_ is the dry season total suspended solids (mg/L), and turbidity is measured in NTU (Nephelometric Turbidity Unit) (Table 1).

**Table 1:** Seasonal variation of water quality of the Halda river.

Parameters	Standard	Dry season (December- February)						Wet season (April- September)						References of standards
1	2	3	4	5	6	1	2	3	4	5	6
DO (mg/L)	>5	5.84	5.85	5.94	4.7	4.1	4.16	3.9	3.5	3.45	3.45	3.1	2.2	Water usable by fishes [41]
DO (in % saturation)		80.66	80.8	82.04	65.13	56.91	57.75	56.86	51.11	50.38	50.38	45.42	32.28	
pH	6.5-8.5	7.33	8.79	7.87	7.86	8.8	8.79	7.2	8.6	8	7.9	8.6	8.65	Water usable by fishes [41]
Turbidity (NTU)	10	82.33	152.94	132	161.35	166.5	157.01	75.43	125.49	78.12	101.5	78.17	130.4	Inland surface water standard from industrial effluents [41]
TSS (mg/L)	150	82.37	116.39	134.74	154.02	165.38	145.45	79.47	85.07	69.08	78.18	101.05	95.68	Inland surface water standard from industrial effluents [41]
TDS (mg/L)	2100	43.01	94.67	91.55	88.34	91.88	61.55	35.01	74.67	70.55	68.37	75.88	55.58	Inland surface water standard from industrial effluents [41]
Ammonia (mg/L)	1.2	0.07	0.61	1.77	1.45	1.64	2.4	0.05	0.51	1.87	1.25	1.34	2.5	Water used for pisciculture
Chloride (mg/L)	600	8.4	59.25	52.61	61.15	55.99	61.8	8.5	65.6	56.3	60.15	65.89	59.47	Inland surface water standard from industrial effluents [41]
Calcium (mg/L)	36	54.27	53.77	60	63.77	63.57	66.17	50.27	52.87	55.28	45.77	59.57	58.17	[28]
COD (mg/L)	200	9.54	26.9	137.04	36.96	46.86	159	8.5	20.8	37.04	40.96	33	35	Inland surface water standard from industrial effluents [41]
BOD_5_ (mg/L)	6<	6.2	7.6	15.1	13.4	18.5	15	3.29	5.16	8.25	9.55	11.41	11.21	water usable by fishes [41]
EC (µS/cm)	1200	90.22	184.55	183.27	177.94	182.33	121.16	107	109	106.4	105.1	105	107	Inland surface water standard from industrial effluents [41]
Temperature (°C)	25	32.5	32.5	32.5	32.7	32.8	32.8	35.8	35.9	35.9	34.1	35.1	34.8	Water usable by fishes [41]

Table 2 comparison between observed physicochemical parameters of Halda River water and WHO recommended drinking water standards. From the table, it can be concluded that some of the parameters such as turbidity, BOD₅, COD, ammonia, and TSS were found in higher concentrations than the recommended standards, especially in the dry season [41].

**Table 2:** Comparison of Observed Water Quality Parameters with WHO Standards.

Parameters	Unit	Observed Range	WHO Permissible Limit	Status
Temperature	°C	32.8–35.9	≤ 30	Above limit
pH	–	7.2–8.8	6.5–8.5	Slightly above
Dissolved Oxygen (DO)	mg/L	2.2–5.9	≥ 5	Below standard
Biochemical Oxygen Demand (BOD₅)	mg/L	3.29–18.5	≤ 3	Exceeds limit
Chemical Oxygen Demand (COD)	mg/L	8.5–40.96	≤ 10	Exceeds limit
Turbidity	NTU	75.4–166.5	≤ 5	Highly above
Electrical Conductivity (EC)	µS/cm	109–184.55	≤ 300	Within limit
Total Dissolved Solids (TDS)	mg/L	35.01–94.67	≤ 500	Within limit
Total Suspended Solids (TSS)	mg/L	69.08–165.38	≤ 50	Above limit
Ammonia (NH₃-N)	mg/L	0.05–2.50	≤ 0.5	Above limit
Chloride	mg/L	8.4–65.89	≤ 250	Within limit
Calcium	mg/L	45.77–66.17	≤ 75	Within limit

#### 2.3.1 Quality control and instrument calibration.

All field and laboratory measurements were conducted following standardized quality control protocols to ensure data accuracy and consistency. Prior to each sampling campaign, the relevant instruments were calibrated. Two-point calibration with zero-oxygen and air-saturated water standards was used to standardize the dissolved oxygen (DO) meter (SD 400 Oxi L, Germany) and the Formazin main standards (range 0–1000 NTU) to standardize the turbidity meter (Hach 2100Q). The pH meter (Hanna HI 9811−5) was standardized by using buffer solutions with pH 4.0, 7.0 and 9.0, while for the calibration of electrical conductivity (EC) 1413 µS/cm potassium chloride (KCl) solution was used. Blanks and duplicate samples (for every ten observations) were used to check the analytical precision in COD and BOD assays. Prior to utilization, all glassware endured acid washing and then rinsed with distilled water. Triplicate analyses were performed to ensure precision, and blanks were employed to check the contamination (if any). Paired sample correlation analysis was performed for cross-validation of seasonal datasets which help to confirm internal consistency and reliability of the findings.

### 2.4 Different Water Quality Index (WQI) applications

International community adopted a variety of WQIs to assess the suitability of surface and groundwater for multipurpose uses including agriculture, drinking, and processing [42]. These indices paint a comprehensive picture of water quality by combining numerous physicochemical factors into a single composite score.

The most well-known water quality indices include the following: the Weighted Arithmetic Water Quality Index (WAWQI) (28 points), the BCWQI (30 points), the CWQI (29 points) (CCME, 2001), the Assigned AWQI (32 points), the MWQI (31 points) (Malaysia), the OWQI (33 points) (Oregon), and the RPI (55 points). To measure the degree of water pollution and its potential impacts on the aquatic environment, each index is based on a different scientific method developed in different climatic and regulatory contexts. When evaluating the Halda River’s overall water quality, this study used seven well-established indices: WAWQI, BCWQI, CWQI, AWQI, MWQI, OWQI, and RPI. To further improve the assessment and validation processes, a data-driven tool called the Method of the Removal Effects of Criteria Water Quality Index (MEREC-WQI) was used. The following sections detail the computational methodologies and methodological formulations used by each index.

### 2.5 Weighted Arithmetic Water Quality Index (WAWQI)

The overall status of water quality can be known from the WAWQI which based on taking a weighted average of the selected parameters and then comparing with the standard limit set for them [43,44]. This index is usually used for the evaluation of the affects caused by waste discharge to the surface water as it is quite easy to calculate and its results can also be compared between locations. Still, it has some weaknesses, such as the subjective choice of weights and the possibility of reducing complex conditions to a single value [23,45–49]. In this study, the WAWQI for the Halda River was calculated using twelve parameters: temperature, electrical conductivity (EC), dissolved oxygen (DO), pH, turbidity, total suspended solids (TSS), total dissolved solids (TDS), ammonia, chloride, calcium, chemical oxygen demand (COD), and biological oxygen demand (BOD₅). The WAWQI is computed using the following equations:


$$\begin{array}{c} WQI=\frac{\sum_{i=1}^{n}q_{i}w_{i}}{\sum_{i=1}^{n}w_{i}} \end{array}$$ (3)

Where q_i_ is the quality rating of the i^th^ water quality parameter and w_i_ is the unit weight of that parameter, as shown below.


$$\begin{array}{c} \sum_{i=1}^{n}w_{i}=1 \end{array}$$ (4)

In addition, q_i_ can be used to determine the correlation between the contaminated water parameter’s value and the standard permitted value.


$$\begin{array}{c} q_{i}=\left(\frac{v_{i}-v_{io}}{s_{i}-v_{io}}\right)\times 100 \end{array}$$ (5)

Where, i^th^ parameter’s measured value (v_i_), ideal value (v_io_), and standard permitted value (s_i_) are defined. With the exception of pH and DO, v_io_ = 0 whenever possible. Specifically, v_io_ = 14.6 mg/L for DO and v_io_ = 7 for pH. There is a negative correlation between w_i_ and the suggested criteria, as shown below.


$$\begin{array}{c} w_{i}=\frac{k}{S_{i}} \end{array}$$ (6)

Where, $\;k=\frac{1}{\sum_{i=1}^{n}\frac{1}{\;s_{i}}}$

Weighted arithmetic index values can be classified from 0 to 100. If the value is near 0, then the water is of high quality. Extremely low water quality is indicated by values near 100. Table 3 shows the results of the physicochemical parameters, with the Bangladesh surface water quality guidelines serving as the standard permitted value for computing the WAWQI. The water can be ranked as excellent (0–25), good (26–50), poor (51–75), very poor (76–100), or above (60–100) if it is not fit for human consumption [49,50].

**Table 3:** Unit weight of WAWQI.

Parameters	Standard	1/S_i_	w_i_
DO (ppm)	5	0.2	0.133105484
DO (in % saturation)			
pH	8.5	0.117647059	0.078297343
EC	300	0.003333333	0.002218425
BOD	6	0.166666667	0.110921237
COD	200	0.005	0.003327637
Temperature	25	0.04	0.026621097
TDS	2100	0.00047619	0.000316918
TSS	150	0.006666667	0.004436849
Ammonia	1.2	0.833333333	0.554606183
Chloride	600	0.001666667	0.001109212
Calcium	36	0.027777778	0.018486873
Turbidity	10	0.1	0.066552742
Total		1.502567694	1
k = 0.66553			

### 2.6 British Columbia Water Quality Index (BCWQI)

The British Columbia Water Quality Index (BCWQI) was developed in 1995 by the British Columbia Ministry of Environment after assessing more than a hundred water bodies to evaluate their compliance with established water quality objectives [21]. This index was designed to measure both how often and by how much the observed values exceed the prescribed standards, offering a systematic way to assess goal attainment and compliance trends over long-term monitoring periods.

This feature also enhances the value of BCWQI for environmental management and policy evaluation [21]. On the other hand, the use of BCWQI is limited by the regionally based weighting and the selection of parameters. In cases where the hydrological or regulatory conditions are significantly different, the BCWQI is not widely applicable. Eight physico-chemical parameters were used (pH, turbidity, ammonia, chloride, total solids, dissolved solids, and total dissolved solids) to calculate the BCWQI, as described in [51]:


$$\begin{array}{c} BCWQI=\sqrt{\frac{\overset{2}{\underset{1}{F}}+\overset{2}{\underset{2}{F}}+(\frac{F_{3}}{3})2}{1.453}} \end{array}$$ (7)

Where, F1, F2 and F3 represents the percent of Parameters that did not meet the water quality objectives (scope), exceedance frequency (frequency) and Maximum amplitude deviations from the objectives (amplitude) respectively. The resulting BCWQI values (scale from 0 to 100) were classified into Unsatisfactory (60–100), Marginal (44–59), Acceptable (18–43), Satisfactory (4–17) and Outstanding (0–3) water quality categories [50].

### 2.7 Canadian Water Quality Index (CWQI)

In 2001, based on the BCWQI experience and CCME pilot studies, the Canadian Council of Ministers of the Environment created the Canadian Water Quality Index (CWQI) to offer an established, structured, and uniform approach for determining water quality in a range of environmental settings. In this study, a total of 12 physico-chemical parameters (temperature, biochemical oxygen demand, chemical oxygen demand, dissolved oxygen, ammonia, chloride, calcium, turbidity, electrical conductivity, pH, total dissolved solids, and total suspended solids) were used to compute the CWQI. With the adoption of three components such as scope (F₁), frequency (F₂), and amplitude (F₃), the water quality is assessed by this index.

Scope (F₁) denotes the percentage of parameters that fail to satisfy criteria at least once over the monitoring period:


$$\begin{array}{c} F_{1}=\left(\frac{Number\;of\;failed\;parameters}{Total\;number\;of\;parameters}\right)\times 100 \end{array}$$ (8)

F_2_, which is a measure of frequency, represents the proportion of tests that do not fulfill the established standards.


$$\begin{array}{c} F_{2}=\left(\frac{Number\;of\;failed\;tests}{Total\;numbre\;of\;tests}\right)\times 100 \end{array}$$ (9)

F_3_ (amplitude measure) is computed in three stages and shows the extent to which test findings differ from the standard standards.

(a) An “excursion” refers to the frequency with which a particular concentration exceeds (or falls below, if the guideline is a minimum) the recommended limit. When the measured value is unable to exceed the threshold:

$$\begin{array}{c} {excursion}_{i}=\left(\frac{{Failed\;Test\;Value}_{i}}{{Objective}_{j}}\right)-1 \end{array}$$ (10)

When the test value must always be equal to or over the threshold:


$$\begin{array}{c} {excursion}_{i}=\left(\frac{{Objective}_{j}}{{Failed\;Test\;Value}_{i}}\right)-1 \end{array}$$ (11)

(b) The aggregate deviation from standards and values is calculated by summing all the deviations of all the tests and then dividing by the total number of tests (including both those that comply with recommendations and those that do not comply with guidelines). The formula provided is utilized to compute the normalized sum of excursions (nse) parameter:

$$\begin{array}{c} nes=\frac{\sum_{i=1}^{n}{excursion}_{i}}{Total\;number\;of\;tests} \end{array}$$ (12)

(c) F_3_ is determined by applying an asymptotic function to the normalized sum of the excursions from recommendations (nse), which is then adjusted to fall within the range of 0–100.

$$\begin{array}{c} F_{3}=\left(\frac{nse}{0.01\times nse+0.01}\right) \end{array}$$ (13)

The index can be calculated by adding the three elements as vectors and applying Pythagoras’ theorem. A squared CCME WQI is the same as a sum of all the factor squares. Here, we think of the index as a three-dimensional space with axes for each component. All three parts of this model are directly related to the index.


$$\begin{array}{c} CWQI=100-\left(\frac{\sqrt{\overset{2}{\underset{1}{F}}+\overset{2}{\underset{2}{F}}+\overset{2}{\underset{3}{F}}}}{1.732}\right) \end{array}$$ (14)

The results were scaled from 0 to 100 using a divisor of 1.732. Based on these scores, the water quality can be categorized into five groups: Poor (0–44), Marginal (45–64), Fair (65–79), Good (80–94) and Excellent (95–100).

Note that the CWQI provides a balanced depiction of both the intensity and occurrence of water quality exceedances, hence it helps to obtain a comparable evaluation throughout the monitoring locations and timeframes [51]. Nevertheless, this index is susceptible to data variability and absent values, hence the reliability may be an issue especially when datasets are incomplete or unevenly distributed [52].

### 2.8 Assigned Water Quality Index (AWQI)

The AWQI is determined by utilizing the standard water criterion and is based on the value of different water characteristics that are meant for usage. An evaluation of the AWQI is carried out in this work using six parameters: EC, pH, turbidity, BOD_5_, and DO. Here is how the AWQI is calculated:

(a) First, a weight (AW_i_) ranging from 1 to 4 from the ref [53] study is assigned to each parameter. The standard criteria are displayed in (Table 4) together with the mean weight values. The weight with the greatest relative importance was 4 and the weight with the least importance was 1.
(b) In this stage, the relative weight (RW) is determined by dividing the sum of given weights by the assigned weight, using the following equation:

**Table 4:** Calculation of AWQI [54–58].

Parameters	Water quality standard	Assigned weight (AW)	Relative weight (RW)
DO (mg/L)	5	4	0.281690141
pH	7.50 (Avg.)	2.1	0.147887324
Turbidity NTU	10	2.4	0.169014085
BOD_5_ (mg/L)	6	3	0.211267606
EC (µS/cm)	1200	2.7	0.190140845
Total		ΣAWi = 14.2	ΣRWi = 1


$$\begin{array}{c} Rw=\left(\frac{{AW}_{i}}{\sum_{i=1}^{n}{AW}_{i}}\right) \end{array}$$ (15)

Where RW is the relative weight, AW is the assigned weight, and n is the total amount of parameters, as shown in Table 4.

(c) This step assigns a quality rating scale (Q_i_) by dividing all the parameters by their normal acceptable criteria, except for pH and DO.

$$\begin{array}{c} Q_{i}=\left(\frac{C_{i}}{S_{i}}\right)\times 100 \end{array}$$ (16)

The following equation is used to determine (QpH, DO) for pH and DO:


$$\begin{array}{c} Q_{pH,DO}=\left(\frac{C_{i}-V_{i}}{S_{i}-V_{i}}\right)\times 100 \end{array}$$ (17)

Where, C_i_ stands for calculating water quality parameters, S_i_ for standard permissible criteria for water quality parameters, and Q_i_ stands for quality rating. The optimal value, v_i_, is determined by taking pH to be 7 and DO to be 14.6. When there are no pollutants in the water, Q_i_ = 0 and when the amount of pollutants is equal to the standard acceptable value, Q_i_ = 100 are the conditions that are applied in Q_i_ and Q_pH, DO_. Therefore, the water is more contaminated the higher the Q_i_ value.

(d) The sub-indices (Sl_i_) for each parameter are calculated in the last phase. The WQI is determined by summing up the total Sl_i_. The equations for calculating the WQI are as follows:


$$\begin{array}{c} {Sl}_{i}={RW\times Q}_{i} \end{array}$$ (18)

$$\begin{array}{c} WQI=\sum_{i=1}^{n}{Sl}_{i} \end{array}$$ (19)

The computed WQI values could be classified as <50 = Excellent; 50–100 = Good; 100–200 = Poor; 200–300 = Very poor; > 300 = Unsuitable [59]. Quick evaluation and management decisions are made possible by the AWQI’s straightforward interpretation and direct connection to local water standards. One major drawback, though, is that it usually only takes a handful of factors into account, which means it can miss some pollutants or new contaminants [54].

### 2.9 Malaysian Water Quality Index (MWQI)

In 1997, the Malaysian Department of Environment created a Water Quality Index (WQI), as demonstrated by [59]. This index is based on six specific factors that measure water quality: DO, BOD_5_, COD, TSS, ammonia, and pH. The study utilizes all six characteristics to ascertain the value of WQI. The formula utilized by Malaysia to calculate the Water Quality Index (WQI) is as follows.


$$\begin{array}{c} WQI=0.22\times SI\;DO+0.19\times SI\;BOD+0.15\times SI\;AN+\;0.12\;\times SI\;pH+\;0.16\;\times SI\;COD+0.16\;\times SI\;SS \end{array}$$ (20)

Where SI is a sub-index value for each of the parameters and the coefficients are the survey answers that determine how much weight to give each element. Following the [60] guidelines will help you find the best-fit equation for determining the different sub-indexes. The MWQI has a scale that goes from 0 to 100. Values close to 0 mean that the water quality is very bad. Values close to 100 means that the water condition is very good. The scores can be put into five groups: very poor (0–25), poor (26–50), fair (51–70), good (71–80), and excellent (81–100) [60]. A primary advantage of the MWQI is its emphasis on aquatic ecosystem health, as it prioritizes parameters directly impacting ecological integrity. A limitation is that it may not fully capture anthropogenic contamination, particularly industrial effluents or emerging pollutants not included among the six core parameters [61].

### 2.10 Oregon Water Quality Index (OWQI)

In the 1970s, the OWQI was created by the Oregon Department of Environmental Quality to evaluate emerging patterns in water quality across many categories [61,62]. Water quality status assessment reports were legally obliged to use it. It was based on the Water Quality Index (WQI) developed by the National Sanitation Foundation and selected properties of water using the Delphi Technique (NSFWQI). In order to classify the water quality factors, we looked at oxygen depletion, eutrophication, dissolved compounds, and health hazards. This study calculates OWQI using five parameters: turbidity, pH, EC, BOD_5_, and DO. When deciding on a basin to use for turbidity calculations, this study takes into account the formula for the Klamath Basin in Oregon, USA. A weighted harmonic squared mean formula, which is the end product of combining NSFWQI and WAWQI, is what gives rise to the OWQI. As an upgrade from NSFWQI and WAWQI, the formula has been proposed by [62]. Following Cude’s description [63], the equation is presented below:


$$\begin{array}{c} OWQI=\sqrt{\frac{n}{\sum_{i=1}^{n}\frac{1}{{{Sl}_{i}}^{2}}}} \end{array}$$ (21)

Where SI is the subindex i of various parameters and n is the total number of subindices. There is a scale from 0 to 100 for the OWQI. Extremely low water quality is indicated by values near zero. Water quality is considered excellent when the value is close to 100. Very poor (0–59), poor (60–79), fair (80–84), good (85–89), and exceptional (90–100) are the five possible rankings for the outcomes [52,63]. The OWQI is advantageous as it reflects both eutrophication and oxygen depletion, providing a more ecologically meaningful assessment than single-parameter indices. One drawback is that it needs to be adjusted locally for areas with tropical or non-temperate climates, since the original design was focused on the climate and hydrology of Oregon [63].

### 2.11 River Pollution Index (RPI)

The River Pollution Index (RPI) assesses pollution levels by analyzing some parameters including dissolved oxygen, biochemical oxygen demand, ammonia, and suspended solids [19,63]. Based on the measured concentration and the associated water quality category, each of the parameter is given a specific score (Sᵢ = 1, 3, 6, or 10), and the following arithmetic mean is adopted to calculated RPI from the allocated scores:


$$\begin{array}{c} RPI=1/4\sum_{i=1}^{4}Si \end{array}$$ (22)

The RPI ranges from 1 to 10, where lower value indicates less pollution. This simple classification system offers easy understanding of river pollution by general public and policymakers as well (Table 5). However, because of its simplicity, this index might miss some other important or emerging pollutants, which could underestimate the total pollution in the river [19].

**Table 5:** River Pollution Index (RPI) [19,64–66].

Items/ranks	Good	Less polluted	Moderately polluted	Highly polluted
BOD_5_ (mg/L)	< 3.5	3.0–4.9	5.0 - 15	> 15
DO (mg/L)	> 6.5	4.6–6.5	2.0–4.5	< 2.0
NH_3_ – N (mg/L)	< 0.5	0.5–0.9	91.0–3.0	> 3.0
SS (mg/L)	< 2.0	20 - 49	50 - 100	> 100
Index scores (Si)	1	3	6	10
Sub – index	< 2	2.0–3.0	3.1–6.0	> 6.0

### 2.12 Multivariate statistical analyses

In order to analyze spatial-temporal correlations and pollution sources, the Principal Component Analysis (PCA) was performed using SPSS v.26. Prior to the analysis, all data were standardized using z-scores. The primary components that explain considerable variance between parameters and WQI values are provided by the PCA. Conversely, the links between individual components and WQI scores are provided by the computed Pearson correlation coefficients.

### 2.13 Method of the Removal Effects of Criteria (MEREC) Water Quality Index (WQI)

The MEREC assesses the relevance of parameters by looking at how the removal each variable from the dataset impacts the outcomes [67]. The following formula determines the i-th parameter’s MEREC contribution:


$$\begin{array}{c} \text{MERECi }=\;\frac{|Q-\overset{\prime }{\underset{i}{Q}}\left|\right|}{Q} \end{array}$$

In this case, Q denotes the original WQI which was determined using all parameters, and Qᵢ′ denotes the WQI which was recalculated following the removal of parameter *i* from the dataset. The MEREC-WQI provides a more objective and repeatable assessment of water quality since it removes subjectivity in weight assignment. However, it necessitates a large dataset with few missing values because missing data could affect the estimation of parameter significance and skew the recalibrated indices. Comparing the use of several WQIs, such as MEREC-WQI and WAWQI, can enhance cross-validation and increase the precision and interpretive reliability of water quality evaluations.

### 2.14 Methodological flow chart

**Fig 2:**
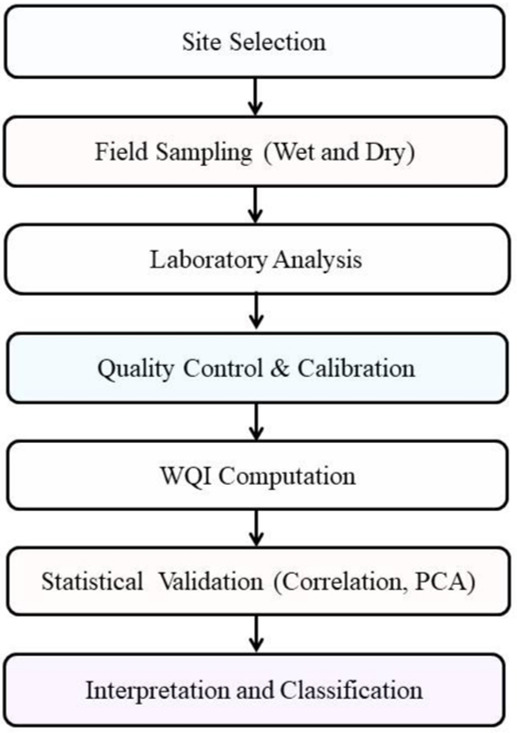
The workflow of the study.

Fig 2 shows the overall workflow of this study with sequential steps:

## 3 Results and discussion

### 3.1 Seasonal variations (dry and wet seasons)

Water quality data were gathered from six segments of the Halda River and analyzed using descriptive statistics, as indicated in Table 6. A significant drop in water quality was indicated by the bulk of the measured physicochemical characteristics being outside of permissible values. Temperature 35.9 °C, pH 8.65, electrical conductivity (EC) 109 µS/cm, biochemical oxygen demand (BOD²) 11.41 mg/L, and turbidity 130.4 NTU were the highest values recorded during the rainy season. The temperature (32.8 °C) and pH (8.8) were slightly lower in the dry season, but the maxima for EC (184.55 µS/cm), BOD₅ (18.5 mg/L), and turbidity (166.5 NTU) were greater. The dry season saw the highest values for other parameters such ammonia (2.5 mg/L), chemical oxygen demand (COD, 40.96 mg/L), dissolved oxygen (DO, 3.9 mg/L), total dissolved solids (TDS, 75.88 mg/L), and total suspended solids (TSS, 101.05 mg/L), whereas the wet season saw the lowest values. These results show the lowest water quality during the dry season, which suggest that the water is unsuitable for human consumption. Reduction of water flow, enhancement of the extraction of water by irrigation, lessened groundwater replenishment, etc. contribute to this seasonal decline [68,69].

**Table 6:** Physico-chemical statistical evaluation of the Halda river.

Parameter	Wet Season					Dry Season				
Maximum	Minimum	Average	Std Dev	Variance	Maximum	Minimum	Average	Std Dev	Variance
Temperature (°C)	35.9	34.1	35.27	±0.73	0.54	32.8	32.5	32.63	±0.15	0.02
pH	8.65	7.2	8.16	±0.57	0.33	8.8	7.33	8.24	±0.64	0.41
EC (μS/cm)	109	105	106.58	±1.48	2.19	184.55	90.22	156.58	±40.67	1654.44
BOD (mg/L)	11.41	3.29	8.15	±3.30	10.90	18.5	6.2	12.63	±4.76	22.68
COD (mg/L)	40.96	8.5	29.22	±12.22	149.38	159	9.54	69.38	±62.53	3910.64
DO (in % saturation)	3.9	2.2	3.27	±0.58	0.34	5.94	4.1	5.10	±0.88	0.77
TDS (mg/L)	75.88	35.01	63.34	±15.66	245.14	94.67	43.01	78.50	±21.23	450.89
TSS (mg/L)	101.05	69.08	84.76	±11.85	140.37	165.38	82.37	133.06	±29.96	897.52
Ammonia (mg/L)	2.5	0.05	1.25	±0.89	0.79	2.4	0.07	1.32	±0.84	0.71
Chloride (mg/L)	65.89	8.5	52.65	±21.95	481.66	61.8	8.4	49.87	±20.60	424.45
Calcium (mg/L)	59.57	45.77	53.66	±5.14	26.45	66.17	53.77	60.26	±5.22	27.25
Turbidity (NTU)	130.4	75.43	98.19	±24.96	623.07	166.5	82.33	142.02	±31.56	996.03

The average values of various metrics for the Halda river are found to be below the national regulations and the Department of Environment (DoE, 1997) permitted standards [28]. In case of dry season, a similar scenario is observed for other rivers in Bangladesh such as the Surma, Titas, Turag-Buriganga, and Balu-Sitalakhya [41, 65,71,72]. This implies that the aquatic ecosystem and the communities nearby the Halda River are in threatened due to the contamination by physicochemical pollutants.

Seasonal variations in the WQI suggest an interplay between human-induced effects and natural hydrological processes. A relatively higher concentration of pollutants during the dry season raises BOD, turbidity, ammonia, and COD levels. Anthropogenic activities including sand mining, extensive farming in the nearby land, residential wastewater discharge from nearby settlements, and the release of industrial waste, all contribute combinedly to the deterioration of water quality of this river [64,70].

In contrast, the wet or rainy season is characterized by greater river discharge and a partial dilution of pollutants, which results in low levels of TSS, COD, and ammonia. However, the WQI scores lie in the low range because of non-point source contamination carried by runoff from agricultural practices and elevated sediment loads [40,71]. In the Halda River, carp usually spawn during the rainy season, when optimal hydrological conditions (e.g., sufficient flow, temperature, and dissolved oxygen) form suitable environment for the survival of eggs and the development of larvae [64]. On the other hand, the ecological integrity of the only natural major carp breeding ground in South East Asia is threatened by the lower dissolved oxygen concentrations, higher turbidity, and higher ammonia concentrations during the dry season, which negatively impact spawning success and larval survival, and create disturbance in the distinctive aquatic biodiversity [17,69] of the River.

### 3.2 Description of Key Water Quality Indicators

To interpret the overall water quality of the Halda River, this study has conducted a complete examination of the major physicochemical parameters. Each parameter provides a separate viewpoint on the ecological status and the degree of human influence on the aquatic ecosystem of the River.

#### 3.2.1 Temperature.

The water temperature showed a varying seasonal pattern in the Halda River, categorized by reduced values (32.50 to 32.80 °C) in the dry season and elevated values (34.10 and 35.90 °C) in the wet season [2,72,73]. Seasonal variations can affect the metabolic processes, solubility of dissolved oxygen, and the biological dynamics within the river system (Table 1).

#### 3.2.2 pH.

Only a modest seasonal fluctuation in the alkalinity of the Halda river water was detected. The readings varied from 7.20 to 8.65 during the wet season and from 7.33 to 8.80 during the dry season. The enhanced alkalinity may be ascribed to the increased bicarbonate concentration, improved photosynthetic activity, and decreased dilution of contaminants in the river water during the dry season. On the other hand, pH values demonstrate the ranges that are acceptable for the majority of freshwater organisms due to the stabilizing impact of increasing runoff (Table 1) [ 39,51].

#### 3.2.3 Electrical Conductivity (EC).

Significant seasonal variations linked to dilution effects were observed in EC. During the dry season, it varied from 90.22 to 184.55 μS/cm, indicating greater ionic concentrations caused by decreased water flow and evaporation. During the rainy season, the range decreased to 105–109 μS/cm, indicating dilution from higher rainfall and runoff. These variations show how dissolved ionic loads and watershed hydrology vary with the seasons (Table 1) [51].

#### 3.2.4 Biochemical Oxygen Demand (BOD).

Changes in organic pollutants were highlighted by seasonal variations in BOD_5_. The BOD_5_ levels ranged from 6.20 to 18.50 mg/L in the dry season, suggesting higher organic loading and lower dilution capability. It decreased to 3.29–11.41 mg/L in the rainy season, which was associated with higher river discharge and improved oxygenation [11,51]. The findings suggest that during drier times, the decomposition of organic materials puts the river ecology under greater stress (Table 1).

#### 3.2.5 Chemical Oxygen Demand (COD).

There were considerable seasonal changes in COD concentrations. Values ranged from 9.54 to 159 mg/L during the dry season, suggesting the buildup of both biodegradable and non-biodegradable organic contaminants in low-flow circumstances [74]. COD fell to 8.50–40.96 mg/L during the wet season, indicating dilution and a reduction in pollutant accumulation. The findings demonstrate that during the dry season, anthropogenic contributions such as residential and agricultural runoff have made a significant impact (Table 1).

#### 3.2.6 Dissolved Oxygen (DO).

Seasonal variations in dissolved oxygen levels were indicative of changes in organic matter input and hydrological conditions. Dissolved oxygen levels ranged from 4.10 to 5.94 mg/L during the dry season and from 2.20 to 3.90 mg/L during the wet season. Increased decomposition of organic materials carried by runoff and limited light penetration due to turbidity may be associated with lower dissolved oxygen levels during the rainy season. The observed seasonal variations point to a degree of stress that aquatic creatures endure, particularly during periods of increased flow and sediment load (Table 1) [75,76].

#### 3.2.7 Total Dissolved Solids (TDS).

The TDS readings showed a moderate seasonal variation, ranging from 35.01 to 75.88 mg/L during the rainy season and from 43.01 to 94.67 mg/L during the dry season. While monsoon runoff contributes to dilution, higher amounts (during the dry season) are most likely caused by evaporation and decreased river flow. Overall, TDS values were continuously low throughout the year, indicating low salinity and little mineralization (Table 1) [72,77].

#### 3.2.8 Total Dissolved Solids (TDS) and Total Suspended Solids (TSS).

Strong seasonal changes linked to sediment transport were seen in TSS. Values during the dry season varied from 82.37 to 165.38 mg/L, indicating slower settling rates, localized disturbances, and erosion. TSS dropped to 69.08–101.05 mg/L during the wet season, most likely as a result of improved mixing and dispersion in spite of increased rainfall. These findings demonstrate that during monsoon flows, suspended sediment burdens are significant yet seasonally moderated (Table 1) [40].

#### 3.2.9 Ammonia-Nitrogen (NH₃–N).

Seasonal variation was seen in ammonia-nitrogen concentrations, which ranged from 0.07 to 2.40 mg/L during the dry season and from 0.05 to 2.50 mg/L during the rainy season. Elevated maxima signify nutrient enrichment resulting from human activities. Ecological risks are presented by the seasonal increases, particularly to fish eggs and larvae (Table 1) [78].

#### 3.2.10 Chloride (Cl⁻).

Chloride concentrations show consistently low values, indicating minimal seasonal change. Wet-season values varied from 8.50 to 65.89 mg/L, while dry-season concentrations ranged from 8.40 to 61.80 mg/L. The somewhat higher maxima during the rainy season most likely reflect improved surface runoff and agricultural drainage. Since chloride is a conservative ion, it can be used as a reliable indication of catchment disturbances and external inputs (Table 1) [39,79].

#### 3.2.11 Calcium (Ca²⁺).

There were minor seasonal differences in calcium concentrations, ranging from 45.77 to 59.57 mg/L during the wet season and from 53.77 to 66.17 mg/L during the dry season. Evaporative concentration and geological leaching under lower flow conditions might result in elevated concentrations during the dry season. Calcium improves aquatic physiology and buffering capacity; low amounts indicate relatively stable water hardness (Table 1) [39].

#### 3.2.12 Turbidity.

There were notable seasonal variations in turbidity. While readings during the wet season ranged from 75.43 to 130.40 NTU, turbidity during the dry season varied from 82.33 to 166.50 NTU. Although turbidity is frequently increased by precipitation, localized disturbances, bank erosion, and less settling under low flow conditions may be the cause of the elevated maximum during the dry season. The quality of fish habitats, light penetration, and primary productivity can all be impacted by increased turbidity (Table 1) [65].

### 3.3 Results of Different Water Quality Indices (WQIs)

Seven established Water Quality Index (WQI) models were used in this study to evaluate the physicochemical status of the Halda River as per the standards set by Bangladesh [33,42]. The spatial and seasonal variations in the water quality of the Halda River can be known, and the major pollution sources that affect the river’s ecology can be easily identified using these indices.

### 3.4 Weighted Arithmetic Water Quality Index (WAWQI)

**Fig 3:**
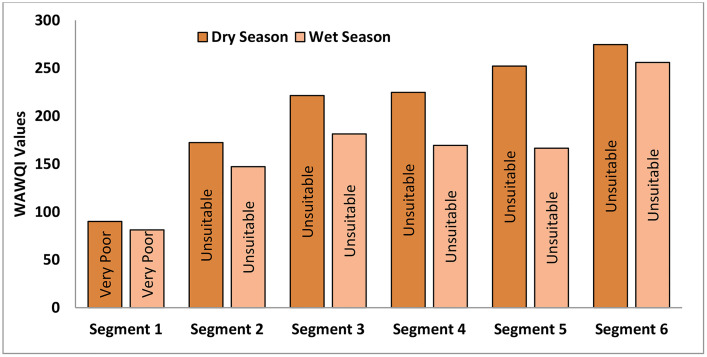
WAWQI of Halda river.

The WAWQI results for six segments of the Halda River during the dry and wet seasons are presented in Fig 3. The highest WAWQI value was recorded **as** 274.62 for the dry season in Segment 6, which was classified as “Unsuitable for fish culture.” The reason behind this poor water quality of Halda river in this segment maybe due to the uncontrolled activities by human living nearby and also industrial activities. Again, segment 1 had the lowest score of 89.93 and it was also classified as “Unsuitable”.

The WAWQI values varies seasonally. There was a slight improvement in values during the wet season than that of dry season due to dilution effect. Segment 6 had the highest value of 255.98 during the wet season. So, the water quality in segment 6 fell into “Unsuitable” category. Segment 1 had the lowest value of 81.19 during the wet season. It also fell in “Poor’ category. Again, most of the water quality parameters values except TDS and TSS were within the allowable limit.

The WAWQI analysis shows that water of Halda river is unsuitable for both domestic and aquaculture use. There are so many factors affecting the deterioration in the water quality of Halda river. Main causes behind this includes agricultural discharge, industrial effluent, domestic waste. This WAWQI also highlighted the need for taking emergency remedial steps to safeguard the river ecology and control the pollution.

### 3.5 British Columbia Water Quality Index (BCWQI)

Table 7 represent the exceedance and variations of selected 8 water quality parameters of Halda river compared with Bangladesh standard [28,41] and BCWQI score.

**Table 7:** BCWQI Computation, Significant values are in bold.

Parameters	Objectives	Wet Season						Dry season					
		1	2	3	4	5	6	1	2	3	4	5	6
pH	6.5-8.5	7.33	8.79	7.87	7.86	8.8	8.79	7.2	8.6	8	7.9	8.6	8.65
Objective exceeded		No	Yes	No	N0	Yes	Yes	No	Yes	No	No	Yes	Yes
Deviation (%)		_	3.29	_	_	3.411	3.294	_	1.16	_	_	1.16	1.73
DO (mg/L)	>5	5.84	5.85	5.94	4.7	4.1	4.16	3.9	3.5	3.45	3.45	3.1	2.2
Objective exceeded		No	No	No	Yes	Yes	Yes	Yes	Yes	Yes	Yes	Yes	Yes
Deviation (%)		_	_	_	6	18	16.8	22	30	31	31	38	56
TDS (mg/L)	< 2100	43.01	94.67	91.55	88.34	91.88	61.55	35.01	74.67	70.55	68.37	75.88	55.58
Objective exceeded		No	No	No	No	No	No	No	No	No	No	No	No
Deviation (%)		_	_	_	_	_	_	_	_	_	_	_	_
TSS (mg/L)	< 150	82.37	116.39	134.74	154.02	165.38	145.45	79.47	85.07	69.08	78.18	101.05	95.68
Objective exceeded		No	No	No	Yes	Yes	No	No	No	No	No	No	No
Deviation (%)		_	_	_	2.61	9.29	_	_	_	_	_	_	_
Ammonia (mg/L)	< 1.2	0.07	0.61	1.77	1.45	1.64	2.4	0.05	0.51	1.87	1.25	1.34	2.5
Objective exceeded		No	No	Yes	Yes	Yes	Yes	No	No	Yes	Yes	Yes	Yes
Deviation (%)		_	_	32.20	17.24	26.83	50	_	_	35.83	4	10.45	52
Chloride (mg/L)	< 13	8.4	59.25	52.61	61.15	55.99	61.8	8.5	65.6	56.3	60.15	65.89	59.47
Objective exceeded		No	Yes	Yes	Yes	Yes	Yes	No	Yes	Yes	Yes	Yes	Yes
Deviation (%)		_	78.06	75.29	78.74	76.78	78.96	_	80.18	76.91	78.39	80.27	78.14
Calcium (mg/L)	< 36	54.27	53.77	60	63.77	63.57	66.17	50.27	52.87	55.28	45.77	59.57	58.17
Objective exceeded		Yes	Yes	Yes	Yes	Yes	Yes	Yes	Yes	Yes	Yes	Yes	Yes
Deviation (%)		33.66	33.05	40	43.55	43.37	45.59	28.39	31.91	34.88	21.35	39.57	38.11
Turbidity (NTU)	< 10	82.33	152.94	132	161.35	166.5	157.01	75.43	125.49	78.12	101.5	78.17	130.4
Objective exceeded		Yes	Yes	Yes	Yes	Yes	Yes	Yes	Yes	Yes	Yes	Yes	Yes
Deviation (%)		87.85	93.46	92.42	93.80	93.99	93.63	86.74	92.03	87.19	90.15	87.21	92.33
F1 = 62.5%, F2 = 56.25%, F3 = 66.10%											BCWQI	63.55	Poor

Turbidity shows the highest variations and exceedance among all other measured parameters. A maximum exceedance of 93.99% is observed in segment 4,5 and 6 during the dry season. Again, segment 6 shows an exceedance of 92.33% in wet season. However, pH shows the lowest exceedance of 1.16%.

The BCWQI value ranges from 0 to 100. Based on this value the water quality can be classified into five categories: exceptional (0–3), decent (4–17), fair (18–43), borderline (44–59), and bad (60–100). The BCWQI values calculated for Halda river was 63.55. According to this value water quality of Halda river is classified as “Poor” category. This result indicates that the water is highly polluted [52]. Though the BCWQI has some limitations in assessing the water quality of a water body [21], these limitations should be considered for proper interpretation.

### 3.6 Canadian water quality index

The use of CWQI is popular for monitoring the trends of environmental change and also for the protection of the vulnerable species of environment [48,80,81]. This tool provides a specific evaluation of water quality of a water body and gives a clear idea to the relevant stakeholder and policy makers regarding the water quality status.

Table 8 shows the CWQI results for the Halda River and a comparison of twelve water quality parameters with the standard limit.

**Table 8:** CWQI calculations. Bold values average exceedance from standard.

Season	Location	Temperature	pH	EC (μS/cm)	BOD (mg/L)	COD (mg/L)	DO (mg/L)	TDS (mg/L)	TSS (mg/L)	Ammonia (mg/L)	Chloride (mg/L)	Calcium (mg/L)	Turbidity (NTU)
Wet	1	35.8	7.2	107	3.29	8.5	3.9	35.01	79.47	0.05	8.5	50.27	75.43
	2	35.9	8.6	109	5.16	20.8	3.5	74.67	85.07	0.51	65.6	52.87	125.49
	3	35.9	8	106.4	8.25	37.04	3.45	70.55	69.08	1.87	56.3	55.28	78.12
	4	34.1	7.9	105.1	9.55	40.96	3.45	68.37	78.18	1.25	60.15	45.77	101.5
	5	35.1	8.6	105	11.41	33	3.1	75.88	101.05	1.34	65.89	59.57	78.17
	6	34.8	8.65	107	11.21	35	2.2	55.58	95.68	2.5	59.47	58.17	130.4
Dry	1	32.5	7.33	90.22	6.2	9.54	5.84	43.01	82.37	0.07	8.4	54.27	82.33
	2	32.5	8.79	184.55	7.6	26.9	5.85	94.67	116.39	0.61	59.25	53.77	152.94
	3	32.5	7.87	183.27	15.1	137.04	5.94	91.55	134.74	1.77	52.61	60	132
	4	32.7	7.86	177.94	13.4	36.96	4.7	88.34	154.02	1.45	61.15	63.77	161.35
	5	32.8	8.8	182.33	18.5	46.86	4.1	91.88	165.38	1.64	55.99	63.57	166.5
	6	32.8	8.79	121.16	15	159	4.16	61.55	145.45	2.4	61.8	66.17	157.01
Standard Value		25	6.5-8.5	300	6	200	5	2100	150	1.2	600	36	10
F1 = 58.33%, F2 = 54.16%, F3 = 54.04%											CCMEWQI	44	poor

During the dry season, the highest exceedances were observed for turbidity (166.5 NTU, segment 5), followed by pH (8.8 mg/L, segment 5), ammonia (2.4 mg/L, segment 5), calcium (66.17 mg/L, segment 6), chloride (61.8 mg/L, segments 4 & 5), and BOD₅ (18.5 mg/L, segment 5). In the wet season, the parameters exceeding the target values included calcium (59.57 mg/L, segment 5), chloride (65.89 mg/L, segments 2 & 5), BOD₅ (11.41 mg/L, segment 5), ammonia (2.5 mg/L, segment 6), and turbidity (125.49 NTU, segment 2).

Higher concentration of turbidity, ammonia and calcium causes a serious pollution in segment 5 during dry season. Significant pollution can also be seen in segment 6 during the wet season. This could also be attributed to the high concentrations of ammonia, chloride, calcium, and turbidity. The elevated level of BOD₅ in Segment 5 in both seasons indicates that unsuitability of the water for the aquaculture. The CWQI assessment for the Halda River produced F₁ (scope) = 58.33%, F₂ (frequency) = 54.16%, and F₃ (amplitude) = 54.04%. The CWQI is classified into five categories based on its value: Excellent (95–100), Good (80–95), Fair (65–79), Marginal (45–64), and Poor (0–44). The CWQI values for the Halda river was found 44.23. So, the water quality of Halda river as per CWQI can be classified as “Poor”. In this category the river water remains severely polluted and aquatic life as well as river ecology become endangered [20,25].

### 3.7 Assigned water quality index

**Fig 4:**
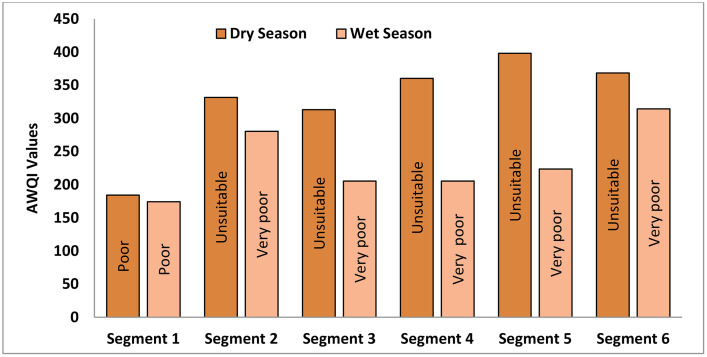
AWQI of Halda river.

Fig 4 represent the AWQI values for six segments of the Halda river in both seasons. Segment 5 recorded the highest AWQI value of 397.99 during the dry season. Again, Segment 1 recorded the lowest AWQI values of 184.32 in the dry season. The water quality in these segments is classified as “Unsuitable” and “Poor”. In the wet season, Segment 6 showed the highest AWQI value of 314.21 and Segment 1 had the lowest AWQI value of 174.13. Water quality in both of these segments fell into “Very Poor” and “Poor” category consistently. So, it is observed from the Fig 4 that, the water quality of Halda river in all six segments remain unsuitable in both seasons which is similar with previous studies [54,59]. The reason behind this water quality deterioration is mainly due to rapid urbanization and climate change.

### 3.8 Malaysian water quality index

**Fig 5:**
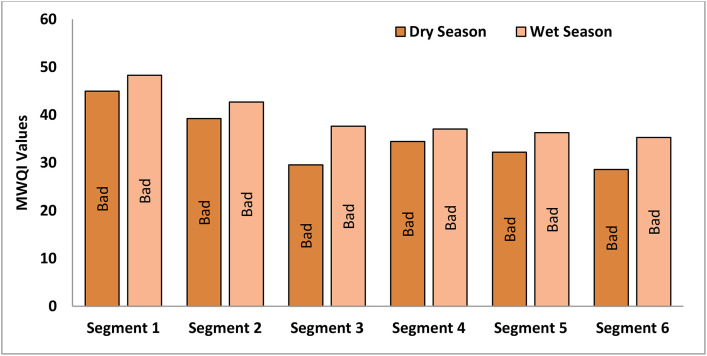
MWQI of Halda river.

Fig 5 shows the MWQI results for the six segments of Halda river in both dry and wet seasons. Based on the MWQI values water quality is classified into five categories: very bad (0–25), bad (26–50), fair (51–70), good (71–80), and exceptional (81–100). Segment 2 showed an MWQI of 39.23 during the dry season and segment 1 also had an MWQI value of 44.98. Water quality in both of these segments fell into the “Bad” category. Again, during the wet season, Segments 1 and 2 were also classified as “Bad,” with MWQI values of 48.30 and 42.70, respectively. The MWQI results for six segments of Halda river in both dry and wet season remain in “Bad” category. This indicates that the water is not suitable for domestic use and can be used for irrigation purpose only [61].

### 3.9 Oregon water quality index

**Fig 6:**
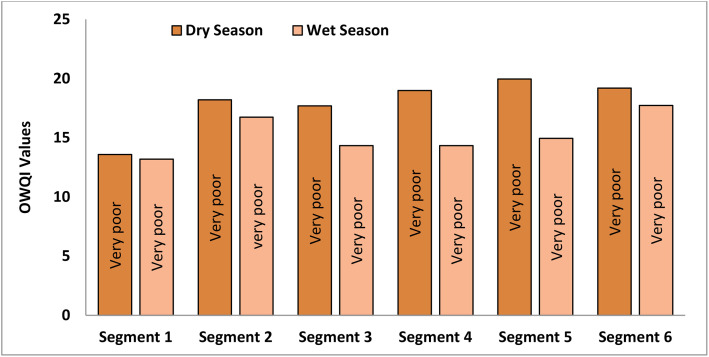
OWQI of Halda river.

Fig 6 shows the results of the OWQI for the six segments of the Halda river during dry and wet season. According to the OWQI values the water quality of a river can be divided into five categories: very poor (0–59), poor (60–79), fair (80–84), good (85–89), and excellent (90–100) [50]. Segment 5 showed the highest OWQI score of 18.97 during the dry season and Segment 1 had the lowest OWQI score of 13.57. Water quality in both of these segments fell into “Very Poor” category. Again, in the wet season, Segment 1 had the lowest OWQI value of 13.19, and Segment 6 had the highest OWQI value of 17.72. The water quality of Halda river in these segments also fell in “Very Poor” category. So, the water quality of Halda river throughout the year in all six segments remain polluted. This raises the issue for taking proper step immediately to protect the river.

### 3.10 MEREC Water Quality Index (MEREC-WQI)

The MEREC (Method of the Removal Effects of Criteria) Water Quality Index provides a more objective method for evaluating water quality by allocating weights to criteria according to their relative significance. In contrast to fixed-weight indices like MWQI or OWQI, the MEREC-WQI assesses the impact of each parameter by examining the alterations in overall water quality upon the exclusion of that parameter. This method facilitates a statistically substantiated ranking of the most significant contaminants.

Table 9 displays the calculated MEREC-WQI values for the Halda River in both the wet and dry seasons. BOD, COD, ammonia, pH, and electrical conductivity (EC) were the most significant metrics, underscoring their pivotal influence on the river’s water quality. In the dry season, the MEREC-WQI attained a value of 82.06, categorizing the water as “Very Poor.” During the wet season, the index fell to 61.11, indicating a “Poor” water quality classification [34].

**Table 9:** Parameter-wise MEREC-WQI Calculation for Halda River.

Parameter	Avg (Wet)	Avg (Dry)	qᵢ Wet	qᵢ Dry	wᵢ MEREC Wet	wᵢ MEREC Dry	Contribution Wet	Contribution Dry
Temperature (°C)	35.27	32.63	88.17	77.57	0.070	0.058	6.17	4.51
pH	8.16	8.24	96.05	98.12	0.084	0.102	8.07	10.00
EC (µS/cm)	106.58	156.58	91.80	97.10	0.086	0.099	7.88	9.61
BOD (mg/L)	8.15	12.63	81.45	126.33	0.099	0.105	8.05	13.26
COD (mg/L)	29.22	69.38	58.43	138.77	0.085	0.107	4.97	14.87
DO (in % saturation)	3.27	5.10	22.54	31.16	0.062	0.059	1.40	1.83
TDS (mg/L)	63.34	78.50	6.33	7.85	0.059	0.051	0.37	0.40
TSS (mg/L)	84.76	133.06	42.38	66.53	0.076	0.084	3.22	5.59
Ammonia (mg/L)	1.25	1.32	83.56	88.22	0.092	0.085	7.68	7.50
Chloride (mg/L)	52.65	49.87	21.06	19.94	0.058	0.046	1.22	0.92
Calcium (mg/L)	53.66	60.26	71.55	80.34	0.067	0.060	4.80	4.82
**MEREC-WQI**							**61.11**	**82.06**

This yearly variation illustrates the concentration effect during the dry period, when diminished river flow, agricultural runoff, and untreated effluents elevate BOD and COD levels. In the wet season, diffusion decreases certain pollutant concentrations; still, ongoing turbidity, ammonia, and organic pollutants sustain overall inadequate water quality.

The elevated BOD and COD levels (13.26 and 14.87 during the dry season) indicate the prevalence of organic pollution sources, including home sewage, agricultural runoff, industrial effluents, and tobacco cultivation in the Halda basin. pH and electrical conductivity exhibited significant influences, indicating chemical changes in the river water resulting from effluents and diminished dilution capability. The comparatively minimal contributions of TDS and DO underscore that, while these metrics reflect overall water quality, they exert less influence than organic load indicators within the MEREC system. Comparable findings were documented by [50] for the Ganga River and by [36] for the Karnaphuli River, validating the efficacy of the MEREC-WQI methodology in tropical river systems.

The MEREC-WQI results align with the other WQI models included in this study. Similar to BCWQI and CWQI, it signifies “Poor to Very Poor” water quality conditions, although its parameter-based weighting enhances the technical rigor and precision of the interpretation. This index specifically highlights the essential contaminants that must be addressed for river restoration and management efforts.

### 3.11 Comparison of different WQIs

**Fig 7:**
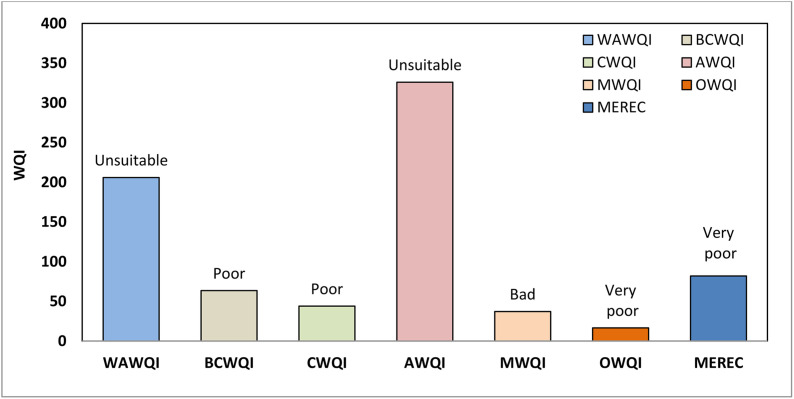
Global Seven WQIs Comparison of Halda river.

Fig 7 shows the comparison between seven water quality indices used for the evaluation of the water quality status of Halda river. Each water quality index described the status of the water quality of Halda river by their own classification criteria and specific approach [20,43]. According to the values of WAQI and AWQI, the water quality of Halda river was categorized as “Unsuitable”. Again, the BCWQI and CWQI results classified the water quality of Halda river as “Poor” category. On the other hand, based on the MWQI and OWQI values the water quality of Halda river fell into the “Bad” category  [82]. The MWQI focuses on the sensitive water quality parameters and follows arithmetic approach [5,6]. The OWQI is provide high precision in case of low value of water quality parameter [83,84].

Among all indices, MEREC-WQI gives the most comprehensive evaluation. A combined weighting of subjective and objective factors, which determines the relative importance of each parameter, was used in this tool [83]. It also provided more detailed information on segment-specific variations and identified key pollution zones.

Therefore, all indices showed that the Halda River’s water quality is deteriorating, but their specific usefulness varies. The WAQI, AWQI, BCWQI, and CWQI are effective for general classification of water quality and trend analysis. On the other hand, the MWQI and OWQI offer more precise identification of pollution levels. MEREC-WQI appears to be the most useful tool for implementing proper river management and pollution control strategies [20,85,83].

### 3.12 River Pollution Index (RPI)

The RPI is a widely used tool to assess the surface water quality. It evaluates four key parameters including DO, BOD₅, TSS, and NH₃–N. Each of parameter is divided into four levels which are used to evaluate the overall pollution level of a water body.

The RPI reference table (Table 4) was used to compare the measured DO and BOD₅ and thus the water quality of Halda river was evaluated. The Mondakini Canal showed an average DO of 3.9 mg/L in a study which represented a moderate level of pollution as per RPI standards [64,76,86]. Similarly, the Madari Canal showed a DO of 4.1 mg/L, which also categorized as moderate pollution level.

### 3.13 Comparison with prior studies

Some previous studies on the use of different WQIs in various rivers across Bangladesh are presented in Table 10. From the table, it is observed that the CWQI and WAWQI were the most commonly used tools for the assessment of water quality in different rivers. Recent studies on the Karnaphuli, Shitalakshya, and Dhaleshwari rivers [10,16,17] showed that the water quality of these rivers **is** classified as “Poor” according to the CWQI. The WAWQI results indicate that the water of these rivers requires pretreatment before use.

**Table 10:** WQI comparisons of various rivers of Bangladesh.

SN	River Name	District	WQI	Rank	Results	References
1	Karnaphuli River	Chittagong	CCME WQI	Poor	Runoff, industrial waste, and municipal trash were all factors in the contamination. You can’t put water for any purpose	[17]
2	Shitalakshya River	Narayanganj	CCME WQI	Poor	Human activity and industrial waste are the primary causes of pollution. No good for farming, drinking, or fishing. When compared to the monsoon season, the water quality in the winter is extremely poor	[10]
			WAWQI	PTBU		
3	Jamuna River	Tangail	AWQI	Unsuitable	Possible sources of contamination include industrial effluents, runoff, anthropogenic activities, and the discharge of municipal wastewater. It is necessary to treat the water properly before using it	[65]
			WAWQI	PTBU		
			OWQI	Very poor		
4	Dhaleshwari River	Tangail	CCME WQI	Poor	Industrial and municipal wastewater, including tannery effluent, sewage, runoff, and others, are the primary sources of pollution. In comparison to the monsoon season, the water quality is significantly lower all winter long	[16]
			WAWQI	PTBU		
5	Turag River	Gazipur	WAWQI	PTBU	Pollutants primarily originate from human activities, sewage overflow, industrial effluent, and runoff	[87]
6	Old Brahmaputra River	Mymensingh	MWQI	Very Polluted	The main sources of pollution are wastewater treatment plants, industrial waste, industrial effluents, and related activities. As a result, this water is suitable only for irrigation purposes.	[18]
7	Halda River	Chittagong	WAWQI	Unsuitable	The sources of pollution in the river were identified using a combination of a structured questionnaire survey and direct field observations. Main contributors were industrial waste (53%), sewage contamination (20%), tobacco farming (13%), a rubber dam (8%), and sand extraction (6%). The Halda River is also the country’s largest natural breeding ground for carp.	Present study
			BCWQI	Poor		
			CCME	Poor		
			AWQI	Unsuitable		
			MWQI	Bad		
			OWQI	Very poor		

Note: PTBU proper treatment is required before use.

In another study, the Brahmaputra River was found to be categorized as “Very Polluted” according to the MWQI. Additionally, a WQI assessment of eleven rivers in Bangladesh found that most rivers were polluted and frequently ranked in the lowest water quality category. The consistent declining of the water quality of these rivers has a linkage with so many factors including waste water discharge from the municipality, industrial effluent, agricultural waste, soil erosion, domestic waste, uncontrolled human activities etc.

These findings from previous studies highlight the necessity for taking immediate steps to protect the river water quality from increasing pollution input.

### 3.14 Correlation and multivariate statistical analysis

A correlation analysis was performed to examine the relationships among different Water Quality Index (WQI) scores, including WAWQI, BCWQI, CWQI, MWQI, and OWQI, alongside essential water quality parameters such as Dissolved Oxygen (DO), Biochemical Oxygen Demand (BOD), Chemical Oxygen Demand (COD), Ammonia Nitrogen (NH₃–N), turbidity, Total Dissolved Solids (TDS), and Total Suspended Solids (TSS). This analysis sought to enhance the understanding of the pollution dynamics in the Halda River.

The findings indicated a significant negative correlation between dissolved oxygen levels and the majority of water quality index scores, implying that reduced oxygen levels are closely linked to diminished water quality. In contrast, turbidity, ammonia, and BOD demonstrated a positive correlation with WQI scores, underscoring their significance as primary factors in the decline of water quality. The observations presented are consistent with earlier research conducted by [40,50], which similarly identified DO, BOD, and turbidity as key factors affecting riverine water quality.

The Pearson correlation study of various WQI indices and principal physico-chemical parameters (Table 10) reveals a robust positive association among the WQI models, signifying their consistent representation of the river’s water quality. Dissolved Oxygen (DO) demonstrates a significant negative association with Water Quality Index (WQI) scores, indicating that diminished oxygen levels are associated with elevated pollution levels. Turbidity, BOD, COD, and ammonia nitrogen (NH₃–N) have a high positive correlation with WQI, indicating that these parameters are the principal contributors to the degradation of water quality in the Halda River [17,64].

Principal Component Analysis (PCA) and Cluster Analysis (CA) were utilized to enhance the comprehension of pollution sources and geographical patterns. PCA indicated that the initial two principal components accounted for a substantial percentage of the overall variation, predominantly influenced by turbidity, BOD, ammonia, and DO. These characteristics indicate the cumulative impact of anthropogenic activities—such as industrial effluent, household wastewater, and agricultural runoff-alongside natural processes, including sedimentation and seasonal fluctuations in river flow.

Cluster analysis categorized the six river segments into two separate categories. Cluster 1 encompassed upstream segments exhibiting relatively superior water quality in the rainy season, whereas Cluster 2 consisted of downstream segments that persistently transported elevated pollutant loads in the dry season. This spatial pattern demonstrates that anthropogenic contamination is concentrated downstream, aligning with the elevated WQI values recorded in these portions [40,71].

The multivariate analyses, corroborated by the correlations in Table 11, elucidate pollution dynamics and seasonal variations, enhancing the WQI findings. These findings provide critical insights for focused river management and pollution reduction methods, enabling the prioritization of locations need immediate attention [50].

**Table 11:** Pearson Correlation Matrix between WQI Scores and Major Physico-Chemical Parameters of the Halda River.

Parameters	WAWQI	BCWQI	CWQI	MWQI	OWQI	DO	BOD	COD	NH₃–N	Turbidity	TDS	TSS
WAWQI	1.000	0.934	0.915	0.902	0.879	−0.842	0.887	0.865	0.813	0.895	0.714	0.796
BCWQI	0.934	1.000	0.942	0.924	0.903	−0.815	0.873	0.852	0.801	0.876	0.705	0.781
CWQI	0.915	0.942	1.000	0.901	0.884	−0.826	0.861	0.834	0.788	0.867	0.691	0.764
MWQI	0.902	0.924	0.901	1.000	0.853	−0.793	0.847	0.812	0.768	0.851	0.676	0.749
OWQI	0.879	0.903	0.884	0.853	1.000	−0.781	0.829	0.801	0.745	0.833	0.661	0.732
DO	−0.842	−0.815	−0.826	−0.793	−0.781	1.000	−0.729	−0.702	−0.689	−0.751	−0.543	−0.628
BOD	0.887	0.873	0.861	0.847	0.829	−0.729	1.000	0.892	0.804	0.874	0.702	0.781
COD	0.865	0.852	0.834	0.812	0.801	−0.702	0.892	1.000	0.781	0.856	0.681	0.749
NH₃–N	0.813	0.801	0.788	0.768	0.745	−0.689	0.804	0.781	1.000	0.813	0.657	0.712
Turbidity	0.895	0.876	0.867	0.851	0.833	−0.751	0.874	0.856	0.813	1.000	0.694	0.768
TDS	0.714	0.705	0.691	0.676	0.661	−0.543	0.702	0.681	0.657	0.694	1.000	0.692
TSS	0.796	0.781	0.764	0.749	0.732	−0.628	0.781	0.749	0.712	0.768	0.692	1.000

Note: Bold correlations (|r| ≥ 0.80) indicate strong relationships.

### 3.15 Implications and Global Relevance of Water Quality Findings

The results of this study carry significant implications for local communities situated along the Halda River, as well as for gaining insights into water quality challenges faced by tropical river systems worldwide. Communities dependent on the Halda River for drinking, irrigation, domestic use, and fisheries face significant impacts from increased levels of BOD, COD, ammonia, and turbidity, especially in the dry season. The pollutants, mainly stemming from agricultural runoff, untreated sewage, and industrial effluents, compromise the river’s physicochemical quality and present potential health risks to residents who consume untreated water [8,65].

High ammonia levels and suspended solids present significant challenges for farmers, contributing to soil salinity, reduced crop productivity, and the long-term degradation of agricultural land. The fisheries of the Halda River, particularly its distinctive carp breeding process, exhibit a high sensitivity to fluctuations in dissolved oxygen and temperature. Additionally, pollution stemming from urban and agricultural activities can interfere with spawning, diminish fish populations, and lead to economic setbacks for local fishermen [88,89]. This study, while not performing a formal health risk assessment or economic valuation, highlights the critical necessity for these analyses to measure community exposure to contaminants and evaluate the economic consequences of diminishing fisheries and agriculture. Incorporating these assessments into upcoming studies would yield a more comprehensive insight into the socioeconomic impacts and bolster the argument for urgent conservation and river management actions [25,50].

From a comprehensive viewpoint, the Halda River exemplifies how swiftly evolving tropical rivers are influenced by anthropogenic factors and seasonal variations. The noted decrease in water quality during the dry season, attributed to reduced flows and elevated pollutant levels, juxtaposed with enhancements during the wet season from rainfall and dilution, reflects trends observed in various tropical and subtropical rivers globally. The impact of agricultural runoff, untreated sewage, and urban discharges on water quality index underscores the difficulties encountered by developing watersheds around the world. Insights gained from the Halda River can inform adaptive water management approaches, aiding in the monitoring and reduction of human impacts while considering seasonal fluctuations [90–97]. These insights play a crucial role in global river health frameworks, offering direction for the management of comparable river systems in Asia and other tropical areas where human activities and hydrological variability significantly impact water quality [98,99].

### 3.16 Limitations of the study

Although the study offers insightful information, there are a few things to keep in mind. Six river segments were included in the sampling, which might not have adequately captured micro-scale differences along the river. Biological or ecological indicators were only partially included in the investigation, which mostly concentrated on physicochemical factors. Short-term pollution events might have gone unnoticed since temporal coverage was restricted to the rainy and dry seasons. Pharmaceuticals, microplastics, and pesticides are examples of emerging pollutants that were not directly measured. Accurate source attribution may be impacted by the lack of industrial effluent data, and the effects of climate change were inferred rather than empirically evaluated. Understanding these restrictions is crucial for analyzing the findings and organizing more thorough future research.

### 3.17 Recommendations for future management

Several recommendations are offered to enhance the water quality and ecosystem health of the Halda River based on the study’s findings. Continuous water quality monitoring systems must be instituted along critical river segments to observe temporal variations. Stringent pollution control techniques are essential for industrial and urban wastewater discharges. Community-based conservation initiatives can mitigate local pollution concerns and enhance awareness. Future evaluations should incorporate biological monitoring, including plankton diversity and fish health biomarkers, to enhance physicochemical analysis. Research on new pollutants and the effects of climate change is crucial for comprehending the long-term implications for water quality and carp reproduction. Ultimately, these findings must be incorporated into policy development and fisheries management strategies to guarantee the sustainable utilization of the Halda River for ecological and socioeconomic advantages.

## 4 Conclusions

This study evaluated the water quality of six segments of Halda river for both wet and dry seasons using widely used seven water quality indices. It also assessed the water quality parameters and their seasonal variations. The findings of this study reveals that all the key water quality parameters measured in this study consistently exceed the allowable limit in almost all six segments throughout the year. The WQI results for six segments of Halda river during both seasons identified the river water quality as “Poor” to “Very Poor”. This is a clear indication that the water of Halda river remain unsuitable for domestic use and aquaculture. This comprehensive analysis using multiple water quality indices identifies a significant knowledge gap of potential impact of consistent water quality degradation on carp reproduction in Bangladesh’s only natural carp-breeding river and economy.

The implications of this study extend beyond the local context, reflecting broader challenges faced by tropical monsoon river systems in developing countries under increasing anthropogenic pressure. The results directly align with the United Nations Sustainable Development Goals, particularly SDG 6 (Clean Water and Sanitation), by evidencing the urgent need for effective water quality management and pollution control strategies. Furthermore, the degradation of water quality and its potential impact on aquatic habitats are closely linked to SDG 14 (Life Below Water), as they threaten biodiversity, disrupt natural breeding grounds, and undermine ecosystem resilience. Immediate actions should be taken to reduce pollutant inputs, regulate industrial activities, follow proper waste management strategies, and promote community-led conservation efforts, which will help restore and maintain the river’s ecological health and economic value.

**Declaration of generative AI and AI-assisted technologies in the writing process**: During the preparation of this work the author(s) used some online service/tools such as Grammarly, ChatGPT 4.0 in order to improve language and readability. After using this tool/service, we reviewed and edited the content as needed and take(s) full responsibility for the content of the publication.

## Supporting information

S1 Fig Graphical abstract. (JPG)

S2 File (DOCX)
