# Supplementary figures and images for: Assessment of water quality of the Halda River using multiple WQI approaches: Implications for Riverine Ecosystem and Sustainable Management
Source: PLoS One. 2026 Jun 18;21(6):e0350672. doi: 10.1371/journal.pone.0350672 (PMC13278588; doi:10.1371/journal.pone.0350672)

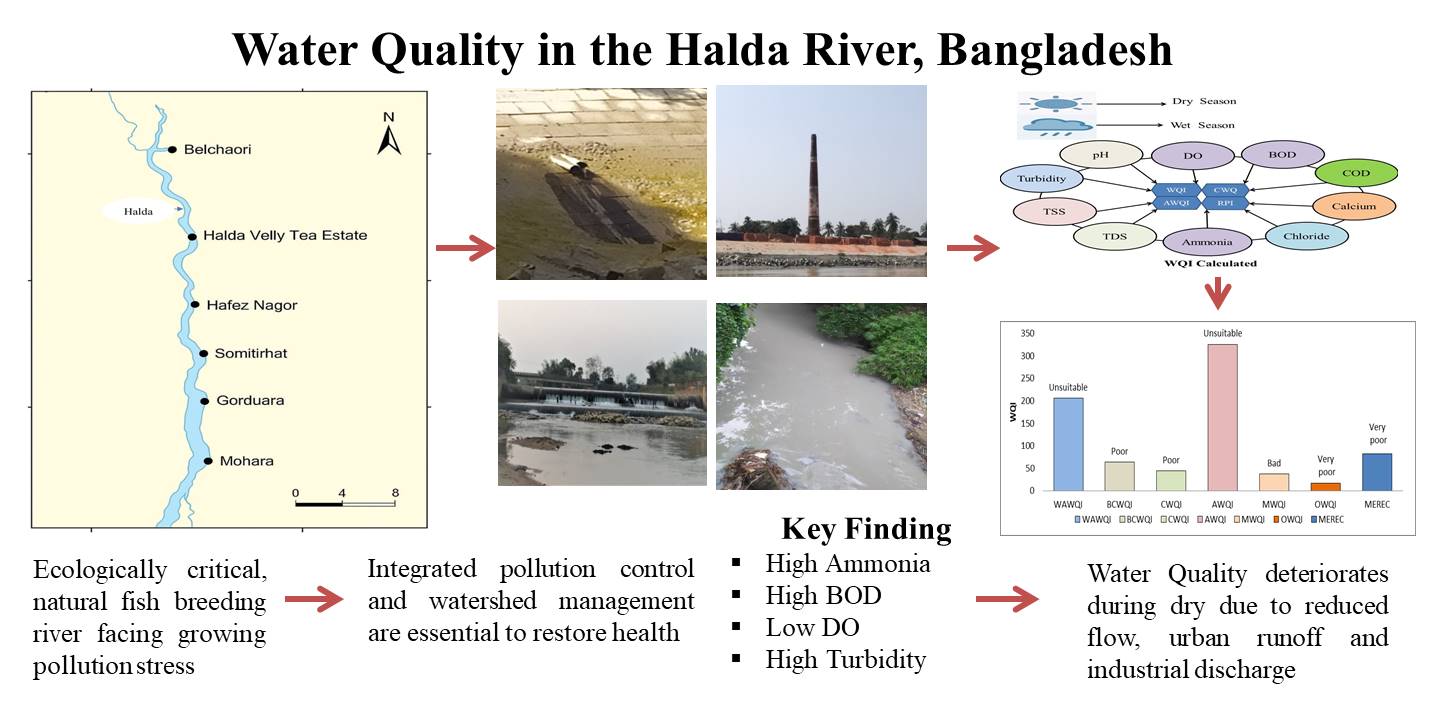

Supplement: S1 Fig — (JPG) [file pone.0350672.s001.JPG]
