# Supplementary material for: Assessment of water quality of the Halda River using multiple WQI approaches: Implications for Riverine Ecosystem and Sustainable Management
Source: PLoS One. 2026 Jun 18;21(6):e0350672. doi: 10.1371/journal.pone.0350672 (PMC13278588; doi:10.1371/journal.pone.0350672)
Supplement: S2 File — (DOCX) [file pone.0350672.s002.docx]

| Paper Title | : | Assessment of the Halda River's Water Quality Using Multiple WQI Approaches: Implications for Riverine Ecosystem and Sustainable Management |
| --- | --- | --- |
| Authors | : | Ahasanul Karim, Muhammad Towhid Moula, Ranjit K. Nath, Masud Rana Rashel, Mh. Mosfeka Chowdhury, Abu Mansur, Md. Masudur Rhaman, Mayeen Uddin Khandaker |

**Supporting information**

Fig 3. WAWQI of Halda river

|  | Dry Season | Wet Season |
| --- | --- | --- |
| Location 1 | 89.93442435 | 81.19751119 |
| Location 2 | 172.2636024 | 147.2869484 |
| Location 3 | 221.4327553 | 181.3659365 |
| Location 4 | 224.8037972 | 169.5057491 |
| Location 5 | 252.2321732 | 166.5869071 |
| Location 6 | 274.6521103 | 255.9820436 |

Fig 4. AWQI of Halda river

|  | Dry Season | Wet Season |
| --- | --- | --- |
| Location 1 | 184.3253169 | 174.1357981 |
| Location 2 | 331.4976819 | 280.3369718 |
| Location 3 | 313.1597946 | 205.3453052 |
| Location 4 | 360.2349648 | 205.3453052 |
| Location 5 | 397.9946655 | 223.4768779 |
| Location 6 | 368.3873944 | 314.2142019 |

Fig 5. MMQI of Halda river

|  | Dry Season | Wet Season |
| --- | --- | --- |
| Location 1 | 44.9808904 | 48.29511507 |
| Location 2 | 39.2319827 | 42.69756374 |
| Location 3 | 29.5380129 | 37.63639679 |
| Location 4 | 34.4273716 | 37.02812103 |
| Location 5 | 32.2263173 | 36.30255569 |
| Location 6 | 28.602299 | 35.26288115 |

Fig 6. OWQI of Halda river

|  | Dry Season | Wet Season |
| --- | --- | --- |
| Location 1 | 13.57664601 | 13.1960524 |
| Location 2 | 18.2070778 | 16.7432665 |
| Location 3 | 17.6963215 | 14.3298746 |
| Location 4 | 18.97985682 | 14.3298746 |
| Location 5 | 19.94980365 | 14.9491431 |
| Location 6 | 19.1934206 | 17.7260882 |

Fig 7. Global Seven WQI Comparison of Halda river

|  | Average WQI |
| --- | --- |
| WAWQI | 205.88 |
| BCWQI | 63.55 |
| CWQI | 44 |
| AWQI | 325.93 |
| MWQI | 37.18 |
| OWQI | 16.57 |
| MEREC | 82.06 |
